# A causal roadmap for generating high-quality real-world evidence

**DOI:** 10.1017/cts.2023.635

**Published:** 2023-09-22

**Authors:** Lauren E. Dang, Susan Gruber, Hana Lee, Issa J. Dahabreh, Elizabeth A. Stuart, Brian D. Williamson, Richard Wyss, Iván Díaz, Debashis Ghosh, Emre Kıcıman, Demissie Alemayehu, Katherine L. Hoffman, Carla Y. Vossen, Raymond A. Huml, Henrik Ravn, Kajsa Kvist, Richard Pratley, Mei-Chiung Shih, Gene Pennello, David Martin, Salina P. Waddy, Charles E. Barr, Mouna Akacha, John B. Buse, Mark van der Laan, Maya Petersen

**Affiliations:** 1 Department of Biostatistics, University of California, Berkeley, CA, USA; 2 TL Revolution, Cambridge, MA, USA; 3 Office of Biostatistics, Office of Translational Sciences, Center for Drug Evaluation and Research, U.S. Food and Drug Administration, Silver Spring, MD, USA; 4 CAUSALab, Department of Epidemiology and Department of Biostatistics, Harvard T.H. Chan School of Public Health, Boston, MA, USA; 5 Department of Mental Health, Johns Hopkins Bloomberg School of Public Health, Baltimore, MD, USA; 6 Biostatistics Division, Kaiser Permanente Washington Health Research Institute, Seattle, WA, USA; 7 Division of Pharmacoepidemiology and Pharmacoeconomics, Brigham and Women’s Hospital, Harvard Medical School, Boston, MA, USA; 8 Division of Biostatistics, Department of Population Health, New York University Grossman School of Medicine, New York, NY, USA; 9 Department of Biostatistics and Informatics, Colorado School of Public Health, University of Colorado Anschutz Medical Campus, Aurora, CO, USA; 10 Microsoft Research, Redmond, WA, USA; 11 Global Biometrics and Data Management, Pfizer Inc., New York, NY, USA; 12 Department of Epidemiology, Mailman School of Public Health, Columbia University, New York, NY, USA; 13 Syneos Health Clinical Solutions, Amsterdam, The Netherlands; 14 Syneos Health Clinical Solutions, Morrisville, NC, USA; 15 Novo Nordisk, Søborg, Denmark; 16 AdventHealth Translational Research Institute, Orlando, FL, USA; 17 Cooperative Studies Program Coordinating Center, VA Palo Alto Health Care System, Palo Alto, CA, USA; 18 Department of Biomedical Data Science, Stanford University, Stanford, CA, USA; 19 Division of Imaging Diagnostics and Software Reliability, Office of Science and Engineering Laboratories, Center for Devices and Radiological Health, U.S. Food and Drug Administration, Silver Spring, MD, USA; 20 Global Real World Evidence Group, Moderna, Cambridge, MA, USA; 21 National Center for Advancing Translational Sciences, Bethesda, MD, USA; 22 Graticule Inc., Newton, MA, USA; 23 Adaptic Health Inc., Palo Alto, CA, USA; 24 Novartis Pharma AG, Basel, Switzerland; 25 Division of Endocrinology, Department of Medicine, University of North Carolina, Chapel Hill, NC, USA

**Keywords:** Causal inference, real-world evidence, sensitivity analysis, simulations, estimands, machine learning

## Abstract

Increasing emphasis on the use of real-world evidence (RWE) to support clinical policy and regulatory decision-making has led to a proliferation of guidance, advice, and frameworks from regulatory agencies, academia, professional societies, and industry. A broad spectrum of studies use real-world data (RWD) to produce RWE, ranging from randomized trials with outcomes assessed using RWD to fully observational studies. Yet, many proposals for generating RWE lack sufficient detail, and many analyses of RWD suffer from implausible assumptions, other methodological flaws, or inappropriate interpretations. The *Causal Roadmap* is an explicit, itemized, iterative process that guides investigators to prespecify study design and analysis plans; it addresses a wide range of guidance within a single framework. By supporting the transparent evaluation of causal assumptions and facilitating objective comparisons of design and analysis choices based on prespecified criteria, the *Roadmap* can help investigators to evaluate the quality of evidence that a given study is likely to produce, specify a study to generate high-quality RWE, and communicate effectively with regulatory agencies and other stakeholders. This paper aims to disseminate and extend the *Causal Roadmap* framework for use by clinical and translational researchers; three companion papers demonstrate applications of the *Causal Roadmap* for specific use cases.

## Introduction

The 21st century has witnessed a dramatic increase in the quality, diversity, and availability of real-world data (RWD) such as electronic health records, health insurance claims, and registry data [1]. In 2016, as part of a strategy to improve the efficiency of medical product development, the US Congress passed the 21st Century Cures Act [2] that mandated the development of US Food and Drug Administration (FDA) guidance on potential regulatory uses of real-world evidence (RWE) – defined as “*clinical* evidence about the usage and potential benefits or risks of a medical product derived from analysis of RWD” [3]. Internationally, stakeholders including other regulatory agencies, industry, payers, academia, and patient groups have also increasingly endorsed the use of RWE to support regulatory decisions [4,5]. Study designs that use RWD to generate RWE (referred to below as RWE studies) include pragmatic clinical trials, externally controlled trials or hybrid randomized-external data studies, and fully observational studies [6–8].

There are multiple motivations for using RWD in a study. First, RWD has long been used in postmarket safety surveillance to uncover the presence of rare adverse events not adequately evaluated by phase III randomized controlled trials for reasons including strict eligibility criteria, strict treatment protocols, limited patient numbers, and limited time on treatment and in follow-up [9]. Second, recent drug development efforts have more commonly targeted rare diseases or conditions without effective treatments [10]. RWD can be useful in such contexts when it is not practical to randomize enough participants to power a standard randomized trial or when there is an ethical imperative to minimize the number of patients assigned to the trial control arm [11,12]. RWD was also highly valuable during the COVID-19 pandemic; observational studies reported timely evidence on vaccine booster effectiveness [13,14], the comparative effectiveness of different vaccines [15], and vaccine effectiveness during pregnancy [16].

Despite the many ways in which RWE may support policy or regulatory decision-making, the prospect of erroneous conclusions resulting from potentially biased effect estimates has led to appropriate caution when interpreting the results of RWE studies. One concern is data availability; data sources might not include all relevant information for causal estimation even in randomized studies that generate RWE. Another concern is lack of randomized treatment allocation in observational RWE. These issues create challenges for estimating a causal relationship outside of the “traditional” clinical trial space.

In an attempt to guide investigators toward better practices for RWE studies, there has been a proliferation of guidance documents and framework proposals from regulatory agencies, academia, and industry addressing different stages of the process of RWE generation [3,5,17–23]. Yet incoming submissions to regulatory agencies lack standardization and consistent inclusion of all information that is relevant for evaluating the quality of evidence that may be produced by a given RWE study [20]. To address this gap between guidance and implementation and to discuss perspectives from regulatory and federal medical research agencies, industry, academia, trialists, methodologists, and software developers, the Forum on the Integration of Observational and Randomized Data (FIORD) meeting was held in Washington, D.C. November 17–18, 2022. FIORD participants discussed their experiences with RWE guidance and best practices, as well as steps that could be taken to help investigators follow available guidance. Specifically, participants determined the need for a unifying structure to assist with specification of key elements of a design and analysis plan for an RWE study, including both the statistical analysis plan (SAP) and additional design elements relevant for optimizing and evaluating the quality of evidence produced.

The *Causal Roadmap* [24–30] (hereafter, the *Roadmap*) addresses this need because it is a general, adaptable framework for causal and statistical inference that is applicable to all studies that generate RWE, including studies with randomized treatment allocation and prospective and retrospective observational designs. It is consistent with existing guidance and makes key steps necessary for prespecifying RWE study design and analysis plans explicit. The *Roadmap* includes steps of defining a study question and the target of estimation, defining the processes that generate data to answer that question, articulating the assumptions required to give results a causal interpretation, selecting appropriate statistical analyses, and prespecifying sensitivity analyses. Following the *Roadmap* may lead to either (1) specification of key elements of a study design and analysis plan that is expected to generate high-quality RWE or (2) an evidence-based decision that an RWE study to generate the required level of evidence is not currently feasible, with insights into what data would be needed to generate credible RWE in the future.

The goal of this paper is to disseminate the *Causal Roadmap* to an audience of clinical and translational researchers. We provide an overview of the *Roadmap*, including a list of steps to consider when proposing studies that incorporate RWD. Members of the FIORD Working Groups also provide three case studies as companion papers demonstrating application of the *Roadmap*, as described in Table 1.

**Table 1.** Companion papers demonstrating use of the *Roadmap*

Case study	Context	Roadmap steps emphasized
Sentinel System and Scalable Phenotyping	Drug safety and monitoring	Outcome-blind^†^ simulations to guide estimator prespecification and machine learning plus natural language processing to enhance identifiability
Nifurtimox for Chagas Disease	Randomized trial infeasible	Sensitivity analysis and defining the plausible causal gap
Semaglutide and Cardiovascular Outcomes	Secondary indications	Application of the *Roadmap* to a hybrid randomized-RWD study and comparison of study designs

^†^We use outcome-blind to mean without information on the observed treatment–outcome association.

## Overview of the Causal Roadmap for Clinical and Translational Scientists

**Figure 1.**
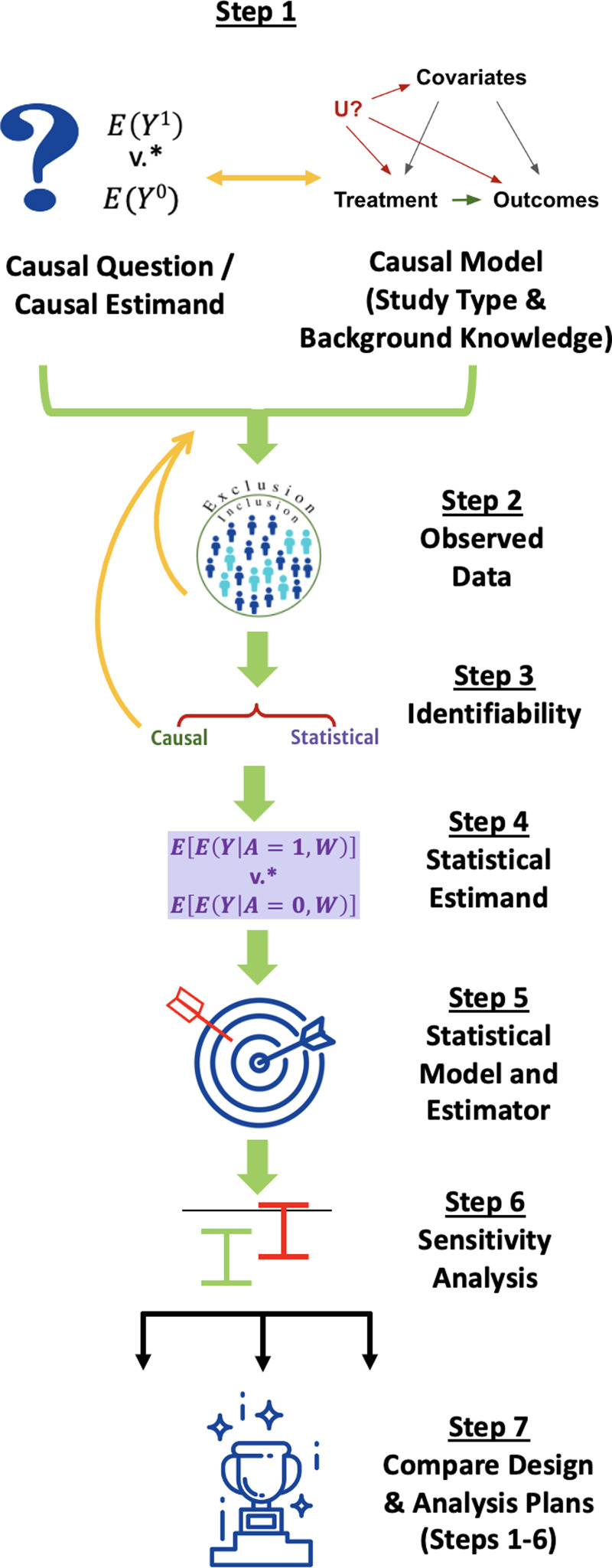
The *Causal Roadmap.* *The contrast of interest may be additive (e.g., risk difference) or multiplicative. (e.g., relative risk).

We walk through the steps of the *Roadmap*, depicted in Fig. 1, explaining their execution in general terms for simple scenarios, why they are important, and why multidisciplinary collaboration is valuable to accomplish each step. The *Roadmap* does not cover all the steps necessary to write a protocol for running a prospective study but instead specifies an explicit process for defining the study design itself, including information that is relevant for evaluating the quality of RWE that may be generated by that design. We suggest that following the *Roadmap* can help investigators generate high-quality RWE to answer questions that are important to patients, payers, regulators, and other stakeholders.

A century’s worth of literature has contributed to the concepts described in the *Roadmap*. Several books explain nuances of these concepts [24,31–36]. The current paper is not a comprehensive introduction but rather aims to describe a structured approach that can support the generation of high-quality evidence.

### Step 1: Causal Question, Causal Model, and Causal Estimand

The first step involves defining the causal question, causal model, and the causal estimand that would answer the question. To facilitate explanation of these concepts, we start by using frameworks for specifying components of a causal question and estimand to also specify key elements of the causal model (Step 1a) before further elaborating the causal model in Step 1b.

### Step 1a: Define the Causal Question and Causal Estimand

Many causal questions start with the objective of estimating the effect of an exposure (e.g., a medication or intervention) on an outcome. Building on decades of research in the careful conduct of randomized and observational studies [36–40], both the International Council for Harmonisation of Technical Requirements for Pharmaceuticals for Human Use (ICH) E9(R1) [41] and Target Trial Emulation [17,32,42,43] frameworks prompt investigators to define components of a causal question and estimand. The causal estimand is a mathematical quantity that represents the answer to the causal question (Table 2).

**Table 2.** Components of a causal question and estimand per ICH E9(R1) [41] and target trial emulation [17]

ICH E9(R1) attribute	Target trial emulation protocol component	Explanation	Related notation in this paper
Population	Eligibility criteria	Inclusion and exclusion criteria, including dates of eligibility, for the potential study population	Measured baseline characteristics^†^: *W*
Treatment	Treatment strategies	The ideal hypothetical intervention(s) of interest in each arm of the target trial, including what treatment or exposure or intervention individuals would experience at study baseline and any postbaseline interventions, such as preventing censoring or requiring adherence for a specified duration. It is also important to consider whether there are different versions of treatment (e.g., different versions of the same surgery performed by different surgeons), and which versions would be included in the treatment strategy [107].	Baseline treatment: A, Censoring^††^: C
	Follow-up period	The events that define the starting (e.g., randomization, prescription) and stopping (e.g., outcome, death) points for the observation period	
Variable or end point	Outcome	Outcome of interest, including the time point(s) at which the outcome will be evaluated	Outcome: *Y*
Population summary	Causal contrasts of interest	Causal estimand^†††^: e.g., average treatment effect, causal relative risk, and average treatment effect within prespecified subgroups	See above

^†^Baseline participant characteristics can include additional variables not used to define eligibility criteria. Baseline variables do not completely characterize the population, but for simplicity, we only consider measured baseline characteristics in the notation below.

^††^In the current paper, we focus on interventions on baseline treatment and postbaseline censoring. However, the approach represented extends naturally to treatment strategies that incorporate additional postbaseline interventions (see e.g., Robins and Hernán (2009) [108], Petersen (2014) [28]).

^†††^A mathematical quantity that is a function of potential outcomes (see above).

An example of a question guided by these components might be: *How would the risk of disease progression by 2 years have differed if all individuals who met eligibility criteria had received the drug under investigation (treatment strategy A* = 1*) versus an active comparator (treatment strategy A* = 0*) and no one dropped out of the study (C* = 0*)?* The best (albeit impossible!) way to answer this question would be to evaluate both the potential outcomes [44,45] individuals would have experienced had they received treatment strategy *A* = 1 and not been censored (*Y* ^*a* = 1, *c* = 0^) *and* the potential outcomes the same individuals would have experienced had they received treatment strategy *A* = 0 and not been censored (*Y* ^*a* = 0, *c* = 0^).

A more fully elaborated structural causal model would help us describe the causal pathways that generate these potential outcomes [46]. For now, we simply consider that, if we were able to observe both potential outcomes for all members of our target population, then the answer to our question would be given by the causal risk difference (or “average treatment effect”):

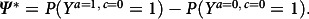




This mathematical quantity – a function of the potential outcomes defined above – is the *causal estimand* of interest in our example. Table 2 lists other examples of causal estimands.

**Importance:** Even though we can only observe at most one potential outcome for each individual [47], and even though it is not possible to guarantee complete follow-up in a real trial, precise definition of the causal question and estimand based on the treatment strategies defined in Table 2 is crucial for specifying a study design and analysis plan to provide the best possible effect estimate. Ultimately, we need a procedure that can be applied to the data to generate an appropriate estimate (e.g., a 5% decrease in risk of disease progression). To assess whether that number provides an answer to our causal question, we must first define mathematically what we aim to estimate.

**Build a Multidisciplinary Collaboration:** Experts in causal inference may come from a variety of fields, including economics, biostatistics, epidemiology, computational science, the social sciences, medicine, pharmacology, and others. They can help to translate a research question into a causal estimand.

### Step 1b: Specify a Causal Model Describing How Data Have Been or Will be Generated

Next, we consider what we know (and do not know) about the processes that will generate – or that have already generated – data to answer this question. First, we consider the type of study (e.g., pragmatic randomized trial, retrospective cohort study). Then, we consider what factors affect the variables that are part of our treatment strategies – found in Table 2 and referred to as intervention variables below – and the outcome in our proposed study. It is also important to consider factors that are affected by intervention and/or outcome variables, such as mediators, colliders, or any study eligibility criteria that are outcome-dependent.

This background knowledge helps to generate the causal model [46]. We specified some key variables in our causal model in Step 1a (in Table 2 and our potential outcomes). Now, we further elaborate our causal model by describing potential causal relationships between these and other important variables. Multiple tools and frameworks can help elicit this information, such as conceptual models and causal graphs (e.g., directed acyclic graphs or single-world intervention graphs) [39,48–51].

**Figure 2.**
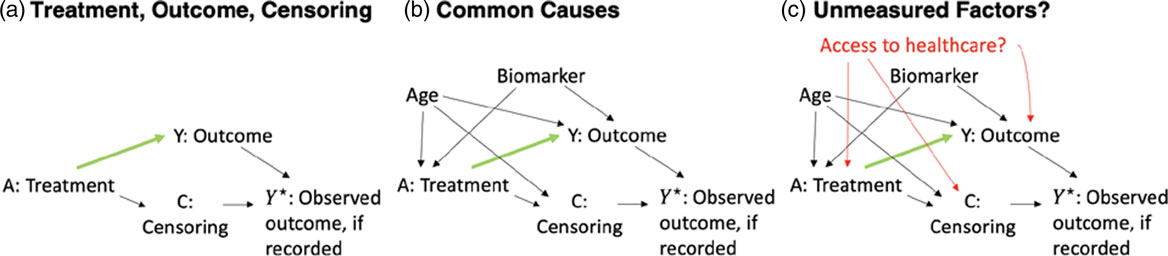
Basic process for generating a causal graph. *Y* ^☆^ is equal to the actual outcome value if it was observed and is missing otherwise.

Fig. 2 gives a simple example of causal graph construction for a prospective observational cohort study, starting with writing down all intervention and outcome variables. When some outcomes are missing, we do not observe the outcome, *Y,* for all participants. Instead, we observe *Y* ^☆^, which is equal to the actual outcome if it was observed and is missing otherwise (Fig. 2a). Arrows denote possible effects of one variable on another.

Then, we attempt to write down factors that might influence (or be influenced by) these variables. Fig. 2b shows two examples (age and a biomarker), though real causal graphs generally include many more variables. In a classic randomized trial, only the randomization procedure affects baseline treatment assignment, whereas in an observational study, participant characteristics or other nonrandomized factors (such as policy or environmental factors) may affect both the treatment/exposure and the outcome. Next, we consider factors that are unmeasured or difficult to measure that might influence treatment, outcomes, or censoring. Fig. 2c shows access to healthcare as an example.

Causal graphs can become much more complicated, especially when working with longitudinal data [52], using proxies for unmeasured variables [53], or combining different data sources [54] (as demonstrated in the case study of Semaglutide and Cardiovascular Outcomes). A carefully constructed causal graph should also include sample selection, competing risks, intercurrent events, and measurement error [32,55]. Examples of causal graph construction are available for a wide variety of study designs including retrospective cohort, cross-sectional, and case–control studies in which selection into the study sample may be affected by the outcome [56,57].

**Importance:** Considering which factors may affect or be affected by intervention variables and outcomes helps to determine whether we can answer our question based on existing data or data that we will collect. The final graph should be our best honest judgment based on available evidence and incorporating remaining uncertainty [32].

**Build a Multidisciplinary Collaboration:** If questions remain about some aspect of this model, such as how physicians decide to prescribe a medication in different practice settings, input from clinicians or other relevant collaborators is obtained before moving on.

### Consider Whether the Causal Question and Estimand (Step 1a) Need to be Modified Based on Step 1b

After writing down our causal model, we sometimes need to change our question [58]. For example, we may have realized that an intercurrent event (such as death) prevents us from observing the outcome for some individuals. As suggested by ICH E9(R1), we could modify the question to consider the effect on a composite outcome of the original outcome or death [41]. ICH E9(R1) discusses other intercurrent events and alternative estimands [41].

### Step 2: Consider the Observed Data

The causal model from Step 1b lets us specify what we know about the real-world processes that generate our observed data. This model can inform what data we collect in a prospective study or help to determine whether existing data sources include relevant information. Next, we consider the actual data we will observe.

Specific questions to address regarding the observed data include the following: How are the relevant exposures, outcomes, and covariates, including those defining eligibility criteria, measured in the observed data? Are they measured differently (including different monitoring protocols) in different data sources or at different time points? Are we able to measure all variables that are important common causes of the intervention variables and the outcome? Is the definition of time zero in the data consistent with the causal question [42]?

**Importance:** After considering these questions, we may need to modify Step 1. For example, if we realize that the data we are able to observe only include patients seen at tertiary care facilities, we may need to change the question (Step 1a) to ask about the difference in the risk of disease progression by two years if all individuals meeting our eligibility criteria *and receiving care at tertiary facilities* received one intervention or the other. Knowledge about factors that affect how variables are measured and whether they are missing should be incorporated in the causal model (Step 1b). Completing this step also helps investigators assess whether the data are fit-for-use [3] and whether we are able to estimate a causal effect from the observed data (discussed in Step 3).

**Build a Multidisciplinary Collaboration:** Clinicians and clinical informaticists can help to explain the way variables are measured in relation to underlying medical concepts or in relation to a particular care setting. Statisticians can help to determine how to match baseline time zero in the observed data with the follow-up period in the causal question.

### Step 3: Assess Identifiability: Can the Proposed Study Provide an Answer to Our Causal Question?

In Step 3, we ask whether the data we observe (Step 2), together with our knowledge about how these data are generated (Step 1b), are sufficient to let us answer our causal question (Step 1a). As described in Step 1a, we cannot directly estimate our causal estimand (which is a function of counterfactual outcomes). Instead, we will express the causal estimand as a function of the observed data distribution (called a statistical estimand, described in Step 4).

The difference between the true values of the statistical and causal estimands is sometimes referred to as the causal gap [27]. If there is a causal gap, even the true value of the statistical estimand would not provide an answer to our causal question. While we can never be certain of the size of the causal gap for studies incorporating RWD and even for many questions using data from traditional randomized trials, we must use our background knowledge to provide an honest appraisal. Causal identification assumptions help us to explicitly state what must be true in order to conclude that the causal gap is zero and that we are thus able to estimate a causal effect using the proposed data. Table 3 lists two examples of identification assumptions with informal explanations of their meaning.

**Table 3.** Examples of identification assumptions

Assumption	Basic explanation of meaning
Exchangeability^*†*^	This assumption is generally true if there are no unmeasured common causes of variables that are part of the treatment strategies (Table 2: e.g., baseline or postbaseline treatment(s), censoring) and the outcome (informally, if there is no unmeasured confounding) and we have not conditioned on a variable that is affected by the treatment variable(s) [32,46].
Positivity	This assumption is true if, for every possible combination of measured confounding variables, individuals with those characteristics have a positive probability of following any of the treatment strategies of interest.

^*†*^Full exchangeability is generally not required if weaker conditions (e.g., mean exchangeability, sequential conditional exchangeability, or others) hold [32].

Exchangeability, in particular, can also be framed in terms of causal graphs [46]. Confounding by unmeasured variables is a widely discussed source of bias in observational studies. Conditioning on a variable that is independently affected by both treatment and the outcome – either by adjusting for that variable in the analysis or by selecting retrospective study participants based on certain values of that variable – may also result in a noncausal association between treatment and outcomes and a statistical estimand that is biased for the true causal effect (i.e., collider bias or selection bias) [32].

Depending on the causal model and question (Step 1), additional assumptions or alternate sets of assumptions may be necessary. For example, if we aim to transport or generalize a causal effect to a new population, we must assume that all values of effect modifiers represented in the target population are also represented in the original study population and that all effect modifiers with different distributions in these two populations are measured [59–63]. Hernán and Robins (2020) [32], among others, provide in-depth discussions of identification assumptions. The three companion papers demonstrate the evaluation of these assumptions.

**Importance:** Considering and documenting the plausibility of the causal identification assumptions helps to determine whether steps can be taken to decrease the potential magnitude of the causal gap. If we conclude that these assumptions are unlikely to be satisfied, then we should consider modifications to Steps 1–2. We may need to limit the target population to those who have a chance of receiving the intervention or evaluate the effect of a more realistic treatment rule to improve the plausibility of the positivity assumption [64,65]. We may need to measure more of the common causes depicted in our causal graph or modify the question to improve the plausibility of the exchangeability assumption [66]. If multiple study designs are feasible, Step 3 can help us to consider which study design is based on more reasonable assumptions [67].

If we know that a key variable affecting treatment and outcomes or censoring and outcomes is not measured, then we generally cannot identify a causal effect from the observed data without measuring that variable or making additional assumptions [17,32,37]. For this and other reasons, many studies analyzing RWD appropriately report statistical associations and not causal effects, though sensitivity analyses (Step 6) may still help to evaluate whether a causal effect exists [68,69]. Nonetheless, if a retrospective study was initially proposed but the causal identification assumptions are highly implausible and cannot be improved using existing data, then investigators should consider prospective data collection to better evaluate the effect of interest.

In general, it would be unreasonable to expect that all causal identification assumptions would be exactly true in RWE studies – or even in many traditional randomized trials that do not utilize RWD due to issues such as informative missingness [32]. Nevertheless, careful documentation of Steps 1–3 in the prespecified analysis plan and in the study report helps not only the investigator but also regulators, clinicians, and other stakeholders to evaluate the quality of evidence generated by the study about the causal effect of interest. Step 3 helps us to specify a study with the smallest causal gap possible. Sensitivity analyses, discussed in Step 6, help to quantify a reasonable range for the causal gap, further aiding in the interpretation of RWE study results.

**Build a Multidisciplinary Collaboration:** Experts in causal inference can aid other investigators in evaluating different causal identification assumptions. For example, reasoning about the exchangeability assumption can become quite complicated if there are multiple intervention variables (e.g., when the treatment varies over time) [39,52]. In such cases, graphical criteria may be used to determine visually from a causal graph whether sufficient variables have been measured to satisfy the exchangeability assumption [39,46,70]. Software programs can also facilitate this process [71,72].

### Step 4: Define the Statistical Estimand

If, after assessing identifiability, we decide to proceed with our study, we aim to define a statistical estimand that is as close as possible to the causal estimand of interest. Recall our causal risk difference for a single time-point intervention and outcome:

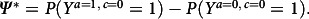




In a simple case where participant characteristics other than our intervention variables and outcome – denoted *W* – are only measured at baseline, then the statistical estimand that is equivalent to the causal effect if all identification assumptions are true is given by:

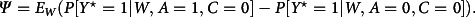




In words, we have rewritten the answer to our causal question (which is defined based on potential outcomes that we cannot simultaneously observe) in terms of a quantity that we can estimate with our data: the average (for our target population) of the difference in risk of our observed outcome associated with the different treatment strategies, adjusted for measured confounders.

**Importance:** The traditional practice of defining the statistical estimand as a coefficient in a regression model has several downsides, even if the model is correctly specified (a questionable assumption, as discussed below) [24]. This approach starts with a tool (e.g., a regression model) and then asks what problem it can solve, rather than starting with a problem and choosing the best tool [73]. For example, the hazard ratio may be estimated based on a coefficient in a Cox regression but does not correspond to a clearly defined causal effect [74–76]. Instead, the *Roadmap* involves choosing a statistical estimand that corresponds to the causal estimand under identification assumptions. We thus specify a well-defined quantity that can be estimated from the observed data and that is directly linked to the causal question.

**Build a Multidisciplinary Collaboration:** Defining a statistical estimand that would be equivalent to the causal effect of interest under identification assumptions is more challenging when there are postbaseline variables that are affected by the exposure and that, in turn, affect both the outcome and subsequent intervention variables [39]. This situation is common in studies where the exposure is measured at multiple time points. In such a situation, statistician collaborators can help to define the statistical estimand using approaches such as the longitudinal g-computation formula [39].

### Step 5: Choose a Statistical Model and Estimator that Respects Available Knowledge and Uncertainty Based on Statistical Properties

The next step is to define a statistical model (formally, the set of possible data distributions) and to choose a statistical estimator. The statistical model should be compatible with the causal model (Step 1b). For example, knowledge that treatment will be randomized (design knowledge that we described in our causal model) implies balance in baseline characteristics across the two arms (with slight differences due to chance in a specific study sample). We could also incorporate knowledge that a continuous outcome falls within a known range or that a dose–response curve is monotonic (e.g., based on prior biological data) into our statistical model. A good statistical model summarizes such statistical knowledge about the form of the relationships between observed variables that is supported by available evidence without adding any unsubstantiated assumptions (such as linearity, or absence of interactions); models of this type are often referred to as semi- or nonparametric or simply realistic statistical models [24].

Given a statistical model, the choice of estimator should be based on prespecified statistical performance benchmarks that evaluate how well it is likely to perform in estimating the statistical estimand [24]. Examples include type I error control, 95% confidence interval (CI) coverage, statistical bias, and precision. Statistical bias refers to how far the average estimate across many samples would be from the true value of the statistical estimand. An estimator must perform well even when we do not know the form of the association between variables in our dataset, and it must be fully prespecified [24].

Many commonly used estimators rely on estimating an outcome regression (i.e., the expected value of the outcome given the treatment and values of confounders), a propensity score (i.e., the probability of receiving a treatment or intervention given the measured confounders), or both. Without knowing the form of these functions, we do not know *a priori* whether they are more likely to be accurately modeled with a parametric regression or a flexible machine learning algorithm allowing for nonlinearities and interactions between variables [24,73,77]. The traditional practice of defaulting to a parametric regression as the statistical estimator imposes additional statistical assumptions, even though they are not necessary. Fortunately, estimators exist that allow for full prespecification of all machine learning and parametric approaches used, data-adaptive selection (e.g., based on cross-validation) of the algorithm(s) that perform best for a given dataset, and theoretically sound 95% confidence interval construction (leading to proper coverage under reasonable conditions) [24].

**Importance:** Effect estimates that are based on incorrectly specified models – such as a main terms linear regression when there is truly nonlinearity or interactions between variables – are biased, and that bias does not get smaller as sample size increases [24]. This bias may result in misleading conclusions. We aim to choose an estimator that not only has minimal bias but also is efficient – thereby producing 95% confidence intervals that are accurate but as narrow as possible – to make maximal use of the data [24].

If, after consideration of the statistical assumptions and properties of the estimators, multiple estimators are considered, then the bias, variance, and 95% CI coverage of all estimators should be compared using outcome-blind simulations that mimic the true proposed experiment as closely as possible [78]. We use the term “outcome-blind” to mean that the simulations are conducted without information on the observed treatment–outcome association in the current study; such simulations may utilize other information from previously collected data or from the current study data if available (e.g., data on baseline covariates, treatment, and censoring) to approximate the real experiment [78]. Simulations conducted before data collection may use a range of plausible values for these study characteristics [79]. As recommended by ICH E9(R1), simulations should also be conducted for cases involving plausible violations of the statistical assumptions underpinning the estimators [41]. Examples of such violations include nonlinearity for linear models or inaccurate prior distributions for Bayesian parameters. For an example of conducting such a simulation, please see the Drug Safety and Monitoring case study.

**Build a Multidisciplinary Collaboration:** Statistician collaborators can help to prespecify an estimator with the statistical properties described above. Resources are increasingly available to assist with prespecification of SAPs based on state-of-the-art estimation approaches. For example, Gruber *et al*. (2022) [80] provide a detailed description of how to prespecify a SAP using targeted minimum loss-based estimation (TMLE) [81] and super learning [77], a combined approach that integrates machine learning to minimize the chance that statistical modeling assumptions are violated [24].

### Step 6: Specify a Procedure for Sensitivity Analysis

Sensitivity analyses in Step 6 attempt to quantify how the estimated results (Step 5) would change if the untestable causal identification assumptions from Step 3 were violated [32,68,82–84]. In contrast, the simulations in Step 5 consider bias due to violations of testable statistical assumptions, which ICH E9(R1) considers as a different form of sensitivity analysis [41]. One mechanism of conducting a causal sensitivity analysis in Step 6 is to consider the potential magnitude and direction of the causal gap; this process requires subject matter expertise and review of prior evidence [68,83–85]. Sensitivity analysis also allows for construction of confidence intervals that account for plausible values of the causal gap [27,68,83–85]. Alternatively, investigators may assess for causal bias using negative control variables [86–87].

The specifics of these methods – and alternative approaches – are beyond the scope of this paper, but the case study of Nifurtimox for Chagas Disease in the companion paper provides an overview of methods for sensitivity analysis, as well as a worked example of using available evidence to assess a plausible range for the causal gap. As discussed in this case study, the method of sensitivity analysis should be prespecified prior to estimating the effect of interest [88]. This process avoids the bias that might occur if experts know the value of the estimate before defining the procedure they will use to decide whether a given shift in that estimate due to bias is reasonable [83].

**Importance:** The process of using prior evidence to reason about likely values of the causal gap helps investigators to assess the plausibility that the bias due to a violation of identification assumptions could be large enough that the observed effect is negated [27,68,69,89]. While the exact magnitude of the causal effect may still not be identified due to known issues such as the potential for residual confounding, if an estimated effect is large enough, we may still obtain credible evidence that an effect exists [69,90]; this was the case in Cornfield et al. (1959)’s seminal sensitivity analysis of the effect of smoking on lung cancer [91]. Conversely, if the anticipated effect size is small and the plausible range of the causal gap is large, the proposed study may not be able to provide actionable information. Considering these trade-offs can help investigators to decide whether to pursue a given RWE study or to consider alternate designs that are more likely to provide high-quality evidence of whether a causal effect exists [69,92].

**Build a Multidisciplinary Collaboration:** If multiple correlated sources of bias are likely, more complex methods of evaluating a plausible range for the causal gap – and collaboration with investigators familiar with these methods – may be required [83].

### Step 7: Compare Alternative Study Designs

*Roadmap* Steps 1–6 help us to specify a study design and analysis plan, including the causal question and estimand, type of study and additional knowledge about how the data are generated, specifics of the data sources that will be collected and/or analyzed, assumptions that the study relies on to evaluate a causal effect, statistical estimand, statistical estimator, and procedure for sensitivity analysis. The type of study described by this design could fall anywhere on the spectrum from a traditional randomized trial to a fully observational analysis. In cases when it is not possible to conduct a traditional randomized trial due to logistical or ethical reasons – or when trial results would not be available in time to provide actionable information – the value of RWE studies is clear despite the possibility of a causal gap [32]. If conducting a randomized trial is feasible, baseline randomization of an intervention (as part of either a traditional or pragmatic trial [93]) still generally affords a higher degree of certainty that the estimated effect has a causal interpretation compared to analysis of nonrandomized data. Yet sometimes, it is feasible to consider multiple different observational and/or randomized designs – each with different potential benefits and downsides.

Consider a situation in which there is some evidence for a favorable risk–benefit profile of a previously studied intervention based on prior data, but those data are by themselves insufficient for regulatory approval for a secondary indication or for clear modification of treatment guidelines. In this context, it is possible that conducting a well-designed RWE study as opposed to a traditional randomized trial alone will shorten the time to a definitive conclusion, decrease the time patients are exposed to an inferior product, or provide other quantifiable benefits to patients while still providing acceptable control of type I and II errors [94–96]. Yet other times, a proposed RWE design may be inferior to alternative options, or one design may not be clearly superior to another. When multiple study designs are considered, outcome-blind simulations consistent with our description of Steps 1–6 can help to compare not only type I error and power but also metrics quantifying how the proposed designs will modify the medical product development process [94]. The case study of Semaglutide and Cardiovascular Outcomes demonstrates how to compare study designs that are based on *Roadmap* Steps 1–6.

**Importance:** A simulated comparison is not always necessary; one study design may be clearly superior to another. Yet often there are trade-offs between studies with different specifications of *Roadmap* Steps 1–6. For example, in some contexts, we may consider augmenting a randomized trial with external data. When comparing the standard and augmented randomized trial designs, there may be a trade-off between (a) the probability of correctly stopping the study early when appropriate external controls are available and (b) the worst-case type I error that would be expected if inappropriate external controls are considered [96]. Another example would be the trade-off between the potential magnitudes of the causal gap when different assumptions are violated to varying degrees for studies relying on alternate sets of causal identification assumptions [67]. Simulated quantification of these trade-offs using prespecified benchmarks can help investigators to make design choices transparent [97].

**Build a Multidisciplinary Collaboration:** Factors to consider when comparing different designs include the expected magnitude of benefit based on prior data and the quality of that data [11], the plausible bounds on the causal gap for a given RWE study, the treatments that are currently available [11], and preferences regarding trade-offs between design characteristics such as type I versus type II error control [97]. Because these trade-offs will be context-dependent [11,97], collaboration with patient groups and discussion with regulatory agencies is often valuable when choosing a study design from multiple potential options.

### A List of Roadmap Steps for Specifying Key Elements of a Study Design and Analysis Plan

Table 4 provides a list of considerations to assist investigators in completing and documenting all steps of the *Roadmap*. Complete reporting of RWE study results should include all prespecified *Roadmap* steps, though information supporting decisions in the final design and analysis plan, such as causal graphs or simulations, may be included as supplementary material. Note that all steps should be prespecified before conducting the study.

**Table 4.** Steps for specifying key elements of a study design and analysis plan using the *Roadmap*

Roadmap step	
1a	Specify the causal question and estimand. ICH E9(R1) attributes: Population, treatment, variable or endpoint, population summary [41] Target Trial Emulation Protocol Components: Eligibility criteria, treatment strategies, follow-up period, outcome, causal contrasts of interest [32]
1b	Specify the causal model (based on background knowledge about the proposed study). Specify the type of study (e.g., traditional randomized trial, retrospective cohort) Document whether censoring, competing risks, or other intercurrent events occurred and factors that may have affected them. Adjust the question as needed.
2	Define the observed data that will be or have been collected. Document how the inclusion/exclusion criteria, treatment variables, outcome(s), and other relevant variables are measured, how time zero is defined, and important differences between data sources.
3	Assess identifiability of the causal estimand from the observed data. Explicitly state the assumptions required for identification, and evaluate their plausibility. Consider modifications to Steps 1–2 to minimize the causal gap. If a retrospective study had been planned but identification assumptions are highly implausible, consider primary data collection or linkage of data from different sources as necessary to ensure relevant information capture for the causal question and estimand.
4	Define the statistical estimand.
5	Specify the statistical model, estimator, and method of confidence interval construction. List the assumptions the proposed estimator and method of confidence interval construction rely upon. Describe the expected statistical bias and variance of the estimator under plausible conditions. If multiple estimators are considered, compare them with outcome-blind simulations based on: statistical bias, variance, confidence interval coverage of the statistical estimand, type I error probability, and power; with plausible violations of model assumptions.
6	Specify the sensitivity analyses. Document the method for defining plausible bounds for the causal gap and/or methods for estimation of the causal gap (e.g., based on negative controls). Provide confidence intervals for the causal effect of interest under the hypothesized size of the causal gap, across the full range of plausible causal gaps.
7	Compare feasible study designs (Steps 1–6) using outcome-blind simulations based on: causal metrics (confidence interval coverage, type I error probability, and power for a causal effect), and metrics to quantify differences in the medical product development process of each design. Include a comparison to a randomized trial if feasible.

## Discussion

The *Roadmap* can help investigators to prespecify design and analysis plans for studies that utilize RWD, choose between study designs, and propose high-quality RWE studies to the FDA and other agencies. We describe the steps of the *Roadmap* in order to disseminate this methodology to clinical and translational scientists. The companion papers presenting case studies on Drug Safety and Monitoring, Nifurtimox for Chagas Disease, and Semaglutide and Cardiovascular Outcomes demonstrate applications of the *Roadmap* and explain specific steps in greater detail.

Past descriptions of the *Roadmap* have largely been targeted to quantitative scientists [24–27,29,30]. In this paper, we focus on intuitive explanations rather than formal mathematical results to make these causal inference concepts more accessible to a wide audience. We emphasize the importance of building a multidisciplinary collaboration, including both clinicians and statisticians, during the study planning phase.

We also introduce an extension of previous versions of the *Roadmap* to emphasize how outcome-blind simulations may be used not only to compare different statistical estimators but also to evaluate different study designs. This extension aligns with the FDA’s Complex Innovative Trial Designs Program guidance for designs that require simulation to estimate type I and II error rates [98] and emphasizes the quantitative comparison of the proposed study to a randomized trial or other feasible RWE designs. The aim of this additional step is to facilitate evaluation of the strengths and weaknesses of each potential approach.

The *Roadmap* aligns with other regulatory guidance documents, as well; these include the FDA’s Framework and Draft Guidance documents for RWE that emphasize the quality and appropriateness of the data [3,99–101] and the ICH E9(R1) guidance on estimands and sensitivity analysis [41]. The *Roadmap* is also consistent with other proposed frameworks for RWE generation. Within the field of causal inference, the *Roadmap* brings together concepts including potential outcomes [44,45], the careful design of nonexperimental studies [35,36,38,40], causal graphs [39,48–51] and structural causal models [46], causal identification [39,46,102], translation of causal to statistical estimands using the g-formula [39], and methods for estimation and sensitivity analysis [24,34,68,77,82,84]. The *Roadmap* is also compatible with frameworks including the Target Trial Emulation framework [17,43], the Patient-Centered Outcomes Research Institute (PCORI) Methodology Standards [19], white papers from the Duke-Margolis Center [18,103], the REporting of studies Conducted using Observational Routinely-Collected health Data (RECORD) Statement [104], the Structured Preapproval and Postapproval Comparative study design framework [105], and the STaRT-RWE template [20]. The purpose of the *Roadmap* is not to replace these – and many other – useful sources of guidance but rather to provide a unified framework that covers key steps necessary to follow a wide range of guidance in a centralized location. Furthermore, while many recommendations for RWE studies list *what* to think about (e.g., types of biases or considerations for making RWD and trial controls comparable), the *Roadmap* aims instead to make explicit a process for *how* to make and report design and analysis decisions that is flexible enough to be applied to any use case along the spectrum from a traditional randomized trial to a fully observational analysis.

With increasing emphasis by regulatory agencies around the world regarding the importance of RWE [5], the number of studies using RWD that contribute to regulatory decisions is likely to grow over time. Yet a recent review of RWE studies reported that “nearly all [reviewed] studies (95%) had at least one avoidable methodological issue known to incur bias” [106]. By following the *Roadmap* steps to prespecify a study design and analysis plan, investigators can set themselves up to convey relevant information to regulators and other stakeholders, to produce high-quality estimates of causal effects using RWD when possible, and to honestly evaluate whether the proposed methods are adequate for drawing causal inferences.
